# Effects of demand-side incentives in improving the utilisation of delivery services in Oyam District in northern Uganda: a quasi-experimental study

**DOI:** 10.1186/s12884-017-1623-y

**Published:** 2017-12-19

**Authors:** William Massavon, Calistus Wilunda, Maria Nannini, Robert Kaos Majwala, Caroline Agaro, Emanuela De Vivo, Peter Lochoro, Giovanni Putoto, Bart Criel

**Affiliations:** 1 Doctors with Africa CUAMM, Aber Hospital, P. O. Box 130, Lira, Uganda; 2 Department of Pharmacoepidemiology, Graduate School of Medicine and Public Health, Kyoto University, Yoshida Konoecho, Sakyoku, Kyoto, Kyoto, 606-8501 Japan; 3 School of Economics and Development, University of Florence, Via delle Pandette, 32, (50127) Florence, Italy; 4 District Health Office, Oyam District Local Government, P. O. Box 30 Loro, Oyam Town Council, Oyam, Uganda; 5 Doctors with Africa CUAMM, Plot 3297 Church Road, P.O. Box 7214, Kampala, Uganda; 6 Doctors with Africa CUAMM, Via San Francisco 126, 35121 Padua, Italy; 7 Institute of Tropical Medicine, Nationalestraat 155, 2000 Antwerp, Belgium; 8 Kampala Capital City Authority, Plot 1-3 Kyagwe Road, P.O. Box 7010, Kampala, Uganda

**Keywords:** Baby kit, Demand-side, Incentives, Maternal and newborn health, Oyam District, Transport vouchers, Uganda

## Abstract

**Background:**

We evaluated the effects and financial costs of two interventions with respect to utilisation of institutional deliveries and other maternal health services in Oyam District in Uganda.

**Methods:**

We conducted a quasi-experimental study involving intervention and comparable/control sub-counties in Oyam District for 12 months (January–December 2014). Participants were women receiving antenatal care, delivery and postnatal care services. We evaluated two interventions: the provision of (1) transport vouchers to women receiving antenatal care and delivering at two health centres (level II) in Acaba sub-county, and (2) baby kits to women who delivered at Ngai Health Centre (level III) in Ngai sub-county. The study outcomes included service coverage of institutional deliveries, four antenatal care visits, postnatal care, and the percentage of women ‘bypassing’ maternal health services inside their resident sub-counties. We calculated the effect of each intervention on study outcomes using the difference in differences analysis. We calculated the cost per institutional delivery and the cost per unit increment in institutional deliveries for each intervention.

**Results:**

Overall, transport vouchers had greater effects on all four outcomes, whereas baby kits mainly influenced institutional deliveries. The absolute increase in institutional deliveries attributable to vouchers was 42.9%; the equivalent for baby kits was 30.0%. Additionally, transport vouchers increased the coverage of four antenatal care visits and postnatal care service coverage by 60.0% and 49.2%, respectively. ‘Bypassing’ was mainly related to transport vouchers and ranged from 7.2% for postnatal care to 11.9% for deliveries. The financial cost of institutional delivery was US$9.4 per transport voucher provided, and US$10.5 per baby kit. The incremental cost per unit increment in institutional deliveries in the transport-voucher system was US$15.9; the equivalent for the baby kit was US$30.6.

**Conclusion:**

The transport voucher scheme effectively increased utilisation of maternal health services whereas the baby-kit scheme was only effective in increasing institutional deliveries. The transport vouchers were less costly than the baby kits in the promotion of institutional deliveries. Such incentives can be sustainable if the Ministry of Health integrates them in the health system.

**Electronic supplementary material:**

The online version of this article (10.1186/s12884-017-1623-y) contains supplementary material, which is available to authorized users.

## Background

Maternal mortality remains a major public-health problem in sub-Saharan Africa (SSA) and in many resource-limited settings. Globally, SSA accounted for 179, 000 (62%) of all estimated 289,000 maternal deaths in 2013. In that year, the maternal mortality ratio (MMR) in low-income countries was 14 times higher than in high-income countries, with SSA having the highest MMR at 510, compared to the global average of 210 per 100,000 live births. The estimated lifetime risk of maternal mortality in high-income countries was 1 in 3400 in comparison to 1 in 52 in low-income countries [1–5].

Maternal mortality has enormous negative effects on child survival, family dynamics, and household economies, as well as national development [6–8]. Consequently, several global, regional, and national initiatives have been employed to reduce maternal mortality and its negative effects, with varying degrees of success. These initiatives include strengthening health systems, implementing safe motherhood strategies, and developing maternal and newborn health networks, which are part of the United Nations Millennium Development Goals (MDGs) and the UN Secretary General’s Strategy, ‘Every woman every child’, among other programmes [9–11]. Reviews, progress reports, and evaluation studies on such initiatives consistently show better outcomes in middle and high-income countries than in low-income countries [12–14], especially SSA. The key reasons often cited for these disparities include weak health systems, low government health expenditures that often translate to catastrophic health expenditures for households, inequities, lack of policies that support the delivery of evidence-based interventions, and lack of access to skilled birth attendants [15, 16].

Uganda is one of the several SSA countries that did not achieve the fifth MDG target by September 2015 [15, 17]. The MMR and institutional delivery rates stood at 435 per 100,000 live births and 52.7%, respectively [18, 19]. In addition to the reasons cited above, many barriers to accessing healthcare exist in Uganda. These include poor geographical access due to distance and transport issues, lack of decision-making power among women, shortage of professional health workers, poor attitudes of some health workers, and preference for traditional birth attendants [2, 4, 5].

Oyam District has one of the highest MMRs (500/100,000 live births) in Uganda [20]. The district is located in a rural post-conflict region in the northern part of the country and had a population of 388, 011 at the time of this study. Over 50% of the population live below the poverty line (US$1.25), more than 70% of the health facilities are health centre IIs, and less than 40% of the population live within 5 km of a health facility. Eighty-nine percent of the pregnant women receive antenatal care (ANC) at least once, but only 48% make four ANC visits (previous recommendation), and 42% use institutional delivery services. The overall rate of caesarean sections is 2.1%, well below the minimum 5% recommended by the WHO [20–24]. This situation has prompted several non-governmental organisations (NGOs) and implementing partners to support healthcare delivery services in the district. If this move is sustained, it could promote institutional deliveries and improve birth outcomes, in line with evidence from literature [25].

The presence of skilled attendants at birth is considered the single most important factor in preventing maternal deaths [26–29], particularly in resource-limited settings. To that end, several innovative approaches that target both demand and supply-side barriers are being implemented. These include incentive-based interventions using strategies, such as conditional cash transfers, clean birth kits and transport voucher schemes. Although most of the studies of such interventions have been conducted in Asia [30–32] and Latin America [33] rather than Africa [34–36], the results have been positive [34, 37] in all the settings.

Few studies have assessed the effects of demand -side incentives, such as a transport voucher scheme, on the proportion of institutional deliveries in Uganda. Nonetheless, there are promising results from one of the preliminary studies [34]. The Ministry of Health (MoH) launched the *Maama* kit initiative in Uganda in 2003 to promote clean and safe deliveries [38]. Under that initiative, *Maama* kits were distributed to pregnant women in mostly rural districts during antenatal visits or community outreach visits [38, 39]. We hypothesised that encouraging pregnant women to deliver at health facilities and providing them with a kit that reduces the cost of newborn care could increase the demand for institutional deliveries. We call this a “baby kit”, contrary to the *Maama* Kit.

We evaluated the effects of providing transport vouchers and baby kits on changes in the number of institutional deliveries, four ANC visits, and postnatal care (PNC) visit. We also measured the proportion of women ‘bypassing’ maternal health services inside their residential sub-counties, in favour of services outside, with respect to four ANC visits, institutional deliveries, and PNC services. As a ‘side objective’, we hypothesised that given the inadequate number and disproportionate distribution of health facilities in the district, this study could help, to some extent, document the extent of ‘bypassing’ in the study’s sub-counties before and during the interventions. We also examined the financial costs of the two interventions in the promotion of institutional deliveries, to scale up to other sub-counties in Oyam District.

## Methods

### Study design and population

This quasi-experimental study evaluated the effects of the two interventions separately: the provision of transport vouchers and baby kits on the utilisation of maternal health services.

The study population consisted of women attending antenatal, delivery and postnatal services at health facilities in the study’s sub-counties.

### Setting

Doctors with Africa CUAMM, an Italian NGO, and hereafter, referred to as CUAMM, implemented the interventions in Oyam District in northern Uganda, from January to December 2014. CUAMM is the main international NGO supporting maternal and newborn healthcare delivery services in Oyam District. This organisation and its operations have been described in previous publications [23, 24, 40, 41]. The papers provide maps of the district and details of the administrative divisions, such as the health sub-districts (HSD), sub-counties, parishes, and villages. Moreover, these papers describe the district’s health system, including the distribution of health facilities and their functional relationships within the district’s health system. Notably, Aber Hospital, a private not-for-profit (PNFP) facility is the only hospital in the district, the single facility capable of providing all emergency obstetric and neonatal care (EmONC) services, and it serves as referral hospital for the district [23]. Table 1 summarises the basic components and services provided at the various levels of the district’s health system.

**Table 1:** Basic components and services at the various levels of the Oyam District Health System

Level	Location	Population	Services provided
^a^Health Centre I (community and Village Health Teams)	Village	1000	Community-based prevention and health-promotion services
^b^Health Centre II	Parish	5000	Prevention, health promotion, and outpatient curative services, outreach
Health Centre III	Sub-County	20,000	Prevention and health-promotion, outpatient curative services, outreach, Maternity, in-patient curative services, and laboratory services
Health Centre IV	County	100,000	Emergency surgery, blood transfusion, and all services provided at HC III level
General or District Hospital (Aber Hospital = PNFP, 178 beds)	District	500,000	Consultations, in-service training, other general services, and research support to community-based healthcare programmes. In addition to services offered at the HC IV level

**Sources:** Health Sector Strategic Plan 2000/01–2004/05, Ministry of Health, Uganda; Health Sector Strategic Plan II 2005/06–2009/10, Ministry of Health, Uganda; Institute for Health Metrics and Evaluation (IHME). Health Service Provision in Uganda: Assessing Facility Capacity, Costs of Care, and Patient Perspectives. Seattle, WA: IHME, 2014

HC, Health Centre. ^a^A HC I is the interface between the formal health system and the community. There are no physical structures, except for a network of Village Health Teams (VHT). The VHTs are non-professional health workers trained to perform several functions. They include health education, disease prevention and health promotion through community sensitisation and mobilisation for various public-health interventions. They are usually residents of the communities and serve as ‘bridges’ between the health facilities and their communities. ^b^Some HC IIs were upgraded and mandated to provide basic maternity services

According to the Uganda National Health Policy [42], there should be a HC III in every sub-county and a HC II in every parish. However, Oyam District has only 6 HC IIIs for the 12 sub-counties, 22 HC IIs for the 63 parishes, a HC IV at Anyeke, and 1 PNFP hospital, for a total of 30 health facilities (Oyam District Health Report 2015, unpublished). Although it is desirable to provide maternity services, mainly at the HC III level and above in the Ugandan health system [43–45], an inadequate number of HC IIIs and their geographical inaccessibility make this provision untenable in Oyam. This situation compelled the district’s local government to upgrade and mandate some HC IIs to provide basic maternity services, in line with existing central government provisions [46, 47].

Furthermore, HCs are distributed unevenly, such that some sub-counties and parishes have two or more health facilities, whereas others have none. This disproportionate distribution of HCs in the sub-counties, coupled with the perceptions of poor quality of care leads to ‘bypassing’ the HCs, a phenomenon whereby residents of poorly served sub-counties seek health services from health facilities in neighbouring sub-counties [48, 49].

Following the presidential election campaign in 2001, Uganda officially abolished user fees in all public-health facilities [50], but that did not lead to zero costs. Patients still pay for transport and sometimes also for medications and commodities at the health facilities because of frequent shortages.

In 2012, CUAMM launched a 5-year programme to improve access to and use of maternal and neonatal health services in the entire district. The programme adopted a mix of demand and supply-side strategies aimed at strengthening the district’s health system and improving the availability, quality, and use of maternal and newborn healthcare services, particularly, skilled attendants at birth. The programme was integrated into the district’s health system, where services are usually free of charge. The key components of that programme were:

- ❖ Budget support to the district’s health office;
- ❖ Quarterly supplies of equipment and drugs and reproductive health commodities to all health facilities in the district;
- ❖ Recruitment of additional qualified human resources for health, mainly nurses and midwives;
- ❖ Training of healthcare workers providing maternal and newborn health services in emergency obstetric and neonatal care, and the provision of incentive packages;
- ❖ Reduced and flat hospital admission fees for maternal and newborn services at Aber Hospital;
- ❖ Free-of-charge caesarean sections at Anyeke HC IV and Aber Hospital;
- ❖ Free ambulance referral services linking the lower- level health facilities to Anyeke HC IV and Aber Hospital;
- ❖ Strengthening of monitoring and evaluation through regular joint support supervisions involving the district’s health authorities and CUAMM staff members;
- ❖ Other measures entailed revitalisation of VHTs to sensitise and mobilise the communities to access and utilise health services; and
- ❖ Retention initiatives, such as performance-based allowances for key staff members who provide comprehensive EmONC services at Aber Hospital and Anyeke HC IV.

The two incentive schemes were implemented within the framework of the programme described above.

### Intervention and control sub-counties and health facilities

In selecting the intervention and control sub-counties, we considered institutional delivery service coverage, ‘bypassing’, the logistical feasibility of implementing the interventions, and the similarity of the health facilities. As the main aim of the interventions was to increase institutional deliveries, the sub-counties with the lowest service coverages were purposively selected. To minimise ‘bypassing’, the control and intervention sub-counties were chosen in such a way that they were separated by a buffer zone. The intervention sub-counties were located in the same HSD to make it logistically feasible to implement the study, given the limited resources available. We also ensured that the control and intervention facilities were fairly similar regarding the levels of care in the health system, infrastructure, services provided, and staffing (Table 1).

Based on the above factors, Alao HC II and Atipe HC II in Acaba sub-county were selected for the transport-voucher intervention. Amwa HC II (Myene sub-county) was selected as a control facility for this intervention. Ngai HC III (Ngai sub-county) was selected for the baby-kit intervention, with Agulurude HC III (Loro sub-county) serving as its control. Each of Acaba and Myene sub-counties has two HC IIs but no HC III. Ngai sub-county has only one HC III, while Loro sub-county has one HC III and two HC IIs.

### Interventions

#### Transport vouchers

The transport-voucher intervention was implemented in Acaba sub-county. The vouchers were given to pregnant women while attending ANC at Alao HC II and Atipe HC II during the intervention period. Some pregnant women in the study’s catchment area learned about the intervention at the time of delivery. To prevent them from feeling being ‘left out’, these women received vouchers to use for PNC services. The voucher allowed women to use any locally available means of transport (motorbike or bicycle) to travel to the health centres for any pregnancy or labour-related condition or emergency, including delivery. The voucher scheme was intended to address geographical inaccessibility. After transport being provided to women, the driver redeemed the voucher at a fixed amount of 10,000 Ugandan Shillings (4 US dollars), at the health facility.

#### Baby kit

To our knowledge, at the time of this study, only Anyeke HC IV in the Oyam District was receiving intermittent supplies of the Ugandan MoH *Maama* kits. That health centre was not included in the study. There are notable differences and minor similarities between the MoH *Maama* kit and the Baby kit that we provided, regarding objectives, contents, and distribution strategies. As shown in the background section, the MoH *Maama* kit initiative was meant primarily to promote clean and safe deliveries. The Maama kit contained a plastic sheet, sterile gloves, razor blades, a cord ligature, a tube of Tetracycline ointment, cotton, sanitary pads and a piece of soap. The kit was offered to pregnant women during antenatal and community outreach visits, which makes the distribution strategy more extensive, compared to our approach.

On the other hand, the baby kits were intended to encourage the use of health facility delivery services by reducing costs related to newborn care. Each baby kit consisted of a plastic basin, a bar of soap, a polythene bag, 1/2 kg of sugar, and a piece of cotton cloth for wrapping the baby. The baby-kit intervention was implemented at Ngai HC III, the only health facility in Ngai sub-county. All pregnant women who delivered at that health centre received baby kits before being discharged.

The project staff visited the intervention facilities monthly to monitor progress, collect the receipts for the vouchers and baby kits, replenish stocks and ensure accountability. Local radio messages and several stakeholder meetings were used to sensitise and mobilise the target communities for the interventions. In addition, the VHTs, community resource persons, and health facilities worked together to raise awareness about the availability of the incentives and how women could access them.

### Study outcomes and cost metrics

The study outcomes included the service coverage of institutional deliveries, four ANC visits, a minimum of one PNC visit, and the proportion of women ‘bypassing’ local health facilities. Institutional delivery was defined as childbirth in a health facility. ANC was defined as the receipt of pregnancy care from skilled providers, while PNC was defined as care at a health facility after childbirth regardless of the place of delivery. Data on these outcomes were extracted from the ANC, delivery, and PNC registers using a standard form designed specifically for this study’s data collection procedures. The form captured monthly summary data on the numbers of ANC visits, institutional deliveries, PNC visits, and referrals all disaggregated by health facility catchment areas. Baseline and endline data were collected throughout 2013 and 2014, respectively. Data on the number of outpatient visits at each participating health facility during these reference periods were also collected.

We calculated costs from the perspective of CUAMM, as the funder of and partner with the district in implementing the interventions. Thus, we did not consider the costs incurred by the users. Data on costs were obtained from the project’s accounting and administrative records for the period January to December 2014. The costs included the values of direct inputs for both intervention arms. This consisted of the cost of each of the items in a baby kit, multiplied by the number of kits distributed, and the total cost of the transport vouchers distributed. Additionally, the costs included labour costs, training costs, sensitisation costs, and administrative support services, notably, the cost of transport for distributing the items and the printing services needed for transport vouchers and receipt books for baby kits. We calculated labour costs by multiplying the number of days in a week spent by drivers and social workers involved in handling vouchers and baby kits by their respective daily net salaries and then annualised the resulting amount. We conducted one joint project start-up training for two staffs from each intervention health facility. Consequently, we allocated training costs in proportion to the number of staff trained for each intervention. We also shared administrative support services costs between the two intervention arms based on the ratio of the institutional deliveries in 2014. Research costs were not included. The costs were handled in Ugandan Shillings (UGX), but for this analysis, we expressed them in US dollars (1 US$ = 2598 UGX as of June 2014) [51]. Details about the cost items are shown in Additional file 1.

### Data analysis

#### Analysis of outcomes

We calculated the service coverage of the four ANC visits, institutional deliveries and PNC as the number of pregnant women who utilised the facility for each of these services in a 12-month period, divided by the annual number of deliveries expected in the area. We performed these calculations separately for baseline and endline data. We estimated the expected number of deliveries by multiplying the crude birth rate for each year by the health facility’s catchment population for the respective year based on official government data. Based on World Bank statistics, we used a crude birth rate (per 1000 population) of 43.5 for 2013 and 43.00 for 2014 [52]. We included women from the study area who had been transferred to other catchment areas for further care in the calculation. For each outcome, we calculated the ‘bypass’ percentage as the proportion of all users from outside the catchment areas of the study’s health facilities. We did not consider women from the study’s catchment areas who might have utilised services in health centres that were not participating in the study.

We performed difference in differences (DID) analyses to estimate the effect of the interventions on each of the outcomes [53]. We calculated the DID estimate of the intervention using the equation: (Y_I2_-Y_I1_)-(Y_c2_-Y_c1_), whereby Y_I2_ is the endline value of a given outcome indicator in the intervention group, Y_I1_ is the baseline value of this indicator in the intervention group, Y_c2_ is the endline value of the indicator in the control group, and Y_c1_ is the baseline value of the indicator in the control group. The DID estimate is thus, the difference in changes over time in the outcomes between the intervention and control groups. Except for the baby-kit and transport-voucher schemes, the health system strengthening programme mentioned above was implemented equally in the control and intervention areas of study. Therefore, any differences in the outcomes between the control and intervention groups can be attributed to the interventions.

#### Analysis of financial costs

The analysis separately compared the two interventions: the transport-voucher system and provision of baby-kit as described in the Methods, to no intervention (the respective control arms). We used institutional deliveries as the main outcome of interest for the cost analysis, and performed a descriptive analysis of the costs for each intervention. Thereafter, we divided the number of institutional deliveries in 2014 in each intervention area by the cost of each intervention to obtain the cost per delivery. We also calculated the incremental cost of each intervention per delivery. To obtain the incremental number of deliveries, we used the formula for the DID analysis presented above, and based the calculations on absolute numbers instead of percentages. We then calculated the incremental cost per delivery by dividing the incremental number of institutional deliveries in each intervention area by the incremental cost of each intervention. All analyses were performed using Microsoft Excel 2010.

## Results

### Service utilisation

**Fig. 1:**
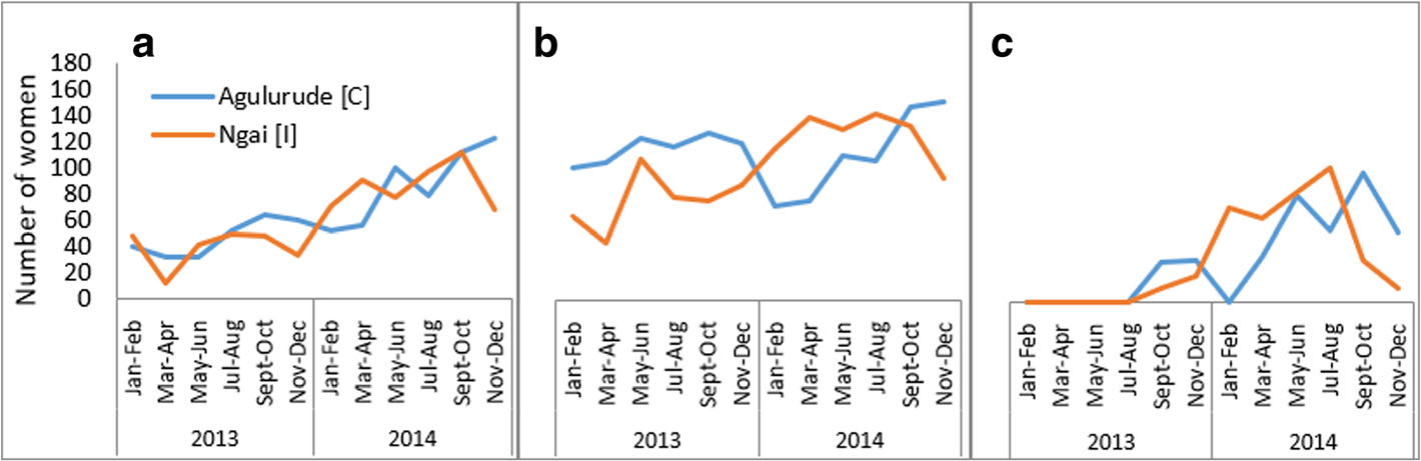
Utilisation of antenatal care (**a**), institutional delivery care (**b**), and postnatal care (**c**) services at baseline and during the baby-kit intervention. The blue line represents the control health centre and the orange line represents the intervention health centre

**Fig. 2:**
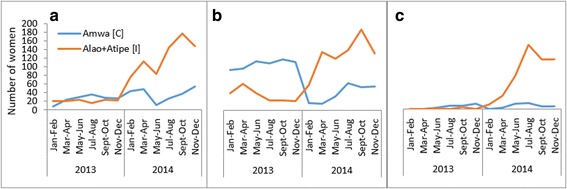
Utilisation of antenatal care (**a**), institutional delivery care (**b**) and postnatal care (**c**) services at baseline and during the transport-voucher intervention. The blue line represents the control health centre and the orange line represents the intervention health centres

The distribution of women who delivered in the intervention and control facilities before, and during the interventions is presented in Table 2. More than 70% of the women who utilised ANC services and had institutional deliveries across the intervention and control facilities, were from the catchment areas of the respective health facilities. We observed similar results concerning PNC, except that the figure was just below 70% at the control health facility for the transport-voucher intervention. The number of clients attending the four ANC visits increased across all the study health facilities after the start of the interventions. In the transport-voucher intervention, the number of institutional deliveries increased at both the intervention and control facilities, although the former showed a greater increase. For the baby kit, the number of deliveries decreased at the control health facility but increased at the intervention facility. The number of postnatal visits rose sharply at all the study health facilities, except the control health facility for the transport-voucher intervention. The trends in the number of ANC visits, institutional deliveries, and PNC visits are presented in Figs. 1 and 2.

**Table 2:** Maternal health services and outpatients’ department users before and during the interventions, Oyam District, 2013–2014

Indicator	Baby kits n (%)				Transport vouchers n (%)			
	Control (Agulurude HC III)		Intervention (Ngai HC III)		Control (Amwa HC II)		Intervention (Atipe + Alao HC IIs)	
	2013	2014	2013	2014	2013	2014	2013	2014
Catchment population	21,832	29,375	24,959	33,582	8649	11,637	25,878	26,235
No. of expected deliveries	950	1263	1086	1444	376	500	1126	1128
Four ANC visits								
n (%) from catchment area	284 (77.0)	525 (72.6)	235 (78.3)	519 (90.4)	152 (70.7)	220 (75.6)	125 (92.6)	738 (89.5)
n (%) bypassing	85 (23.0)	198 (27.4)	65 (21.7)	55 (9.6)	63 (29.3)	71 (24.4)	10 (7.4)	87 (10.5)
Total four ANC visits	369	723	300	574	215	291	135	825
Institutional delivery								
n (%) from catchment area	675 (79.4)	646 (72.2)	407 (80.4)	734 (87.6)	130 (77.8)	249 (81.4)	224 (98.2)	811 (89.9)
n (%) bypassing	175 (20.6)	249 (27.8)	99 (19.6)	104 (12.4)	37 (22.2)	57 (18.6)	4 (1.8)	91 (10.1)
Total deliveries	850	895	506	838	167	306	228	902
PNC visits								
n (%) from catchment area	61 (83.6)	307 (74.7)	29 (100)	348 (94.3)	33 (68.8)	43 (69.4)	7 (100)	520 (93.4)
n (%) bypassing	12 (16.4)	104 (25.3)	0 (0)	21 (5.7)	15 (31.2)	19 (30.6)	0 (0)	37 (6.6)
Total PNC	73	411	29	369	48	62	7	557
Total OPD attendance^a^	20,751	18,607	20,809	17,976	6834	6203	17,169	14,483

*OPD* Outpatients’ department, *ANC* Antenatal care, *PNC* Postnatal care. ^a^OPD attendance is largely driven by malaria in the district. In 2014, there was a district-wide distribution of insecticide-treated bed nets and indoors residual spraying for malaria control, which explain the drop from the 2013 figures

### Effect of the interventions on service utilisation

According to the DID analysis, 30.0% of the expected deliveries that occurred in the catchment area of the baby-kit intervention was associated with the offer of a baby kit (Table 3). This intervention had a negligible effect on the four ANC visits and PNC. On the other hand, the transport vouchers had strong positive effects on the utilisation of all three maternal health services (Table 4). The service coverage of the four ANC visits, institutional deliveries, and PNC in the intervention catchment areas, attributable to the transport vouchers were 60.0%, 42.9%, and 49.2%, respectively.

**Table 3:** Effect of baby kit on the utilisation of maternal health services, Oyam District, 2013–2014

Outcome	Control (Agulurude HC III) % coverage			Intervention (Ngai HC III) % coverage			DID %
	2013	2014	Difference	2013	2014	Difference	
ANC 4	38.9	57.2	18.3	27.6	39.7	12.1	−6.2
Delivery	89.5	70.9	−18.6	46.6	58.0	11.4	30.0
PNC	7.7	32.5	24.8	2.7	25.6	22.9	−2.0

*ANC* Antenatal care, *PNC* Postnatal care, *DID* Difference in differences (analysis). Service coverage was calculated using the information in Table 2. For instance, institutional delivery coverage in 2013 in the control group was obtained by dividing the total number of institutional deliveries in 2013 by the expected number of deliveries in the reference area in 2013 i.e. 850/950*100 = 89.5%

**Table 4:** Effect of transport vouchers on the utilisation of maternal health services, Oyam District, 2013–2014

Outcome	Control (Amwa HC II) % coverage			Intervention (Atipe HC II + Alao HC II) % coverage			DID %
	2013	2014	Difference	2013	2014	Difference	
ANC 4	57.1	58.2	1.1	12.0	73.1	61.1	60.0
Delivery	44.4	61.2	16.8	20.3	80.0	59.7	42.9
PNC	12.8	12.4	−0.4	0.6	49.4	48.8	49.2

*ANC* Antenatal care, *PNC* Postnatal care, *DID* Difference in differences (analysis). Service coverage was calculated using the information in Table 2. For instance, PNC coverage in 2013 in the control group was obtained by dividing the total number of women who received PNC in 2013 by the expected number of deliveries in the reference area in 2013 i.e. 48/376*100 = 12.8%

### ‘Bypassing’

The proportion of women receiving maternal health services at the intervention health facilities, who were not from the facilities’ catchment areas (‘bypassers’), reduced at the health centre that distributed the baby kit, but increased at those that distributed the transport vouchers (Table 5).

**Table 5:** Maternal health services and **‘**bypassing’ analysis, Oyam District, 2013–2014

Outcome	Control			Intervention			DID (%)
	2013	2014	Difference	2013	2014	Difference	
Baby kits intervention							
Four ANC visits bypass	23.0	27.4	4.4	21.7	9.6	−12.1	−16.5
Delivery bypass	20.6	27.8	7.2	19.6	12.4	−7.2	−14.4
PNC bypass	16.4	25.3	8.9	0.0	5.7	5.7	−3.2
Transport vouchers							
Four ANC visits bypass	29.3	24.4	−4.9	7.4	10.5	3.1	8.0
Delivery bypass	22.2	18.6	−3.5	1.8	10.1	8.3	11.8
PNC bypass	31.3	30.6	−0.6	0.0	6.6	6.6	7.2

*ANC* Antenatal care, *PNC* Postnatal care, *DID* Difference in differences (analysis). The percentages of women ‘bypassing’ are from Table 2

### Referrals from lower level health facilities to Anyeke HC IV and Aber hospital

The analysis showed that the interventions increased the proportion of pregnancy and labour-related referrals (i.e. women transferred from study health centres to the district’s referral facilities for further management) by 36.8% (Additional file 2).

### Financial costs of the interventions

Table 6 shows the descriptive analysis of the cost of each intervention per unit institutional delivery, and the incremental cost of each intervention per unit institutional delivery. Direct costs accounted for 57.6% and 65.4% of the total cost in the baby kit and transport voucher arms, respectively. The number of institutional deliveries attributable to the transport vouchers and baby kits were 535 and 287, respectively. The total cost of implementing the transport-voucher system was US$8478.3; whereas it costed the project US$8774.4 to provide baby kits. Hence, the cost per unit institutional delivery was US$10.5 and US$ 9.4 in the baby kit and voucher arms, respectively. The incremental cost per unit increment in the institutional delivery due to the transport-voucher system was US$15.9; the cost for the baby kit was US$30.6.

**Table 6:** Analysis of costs and incremental unit costs associated with the interventions, Oyam District, 2013–2014

	Baby kits			Transport vouchers		
No. of deliveries	838			902		
No. of deliveries attributable to intervention (DID estimate)	287			535		
COSTS	UGX	US$	% of cost	UGX	US$	% cost
Direct costs	13,119,500	5049.85	57.6	14,404,000	5544.26	65.4
Sensitisation costs	3,804,713	1464.48	16.7	4,095,287	1576.32	18.6
Training costs	183,333	70.57	0.8	366,667	141.13	1.7
Labour costs	5,139,168	1978.12	22.5	2,569,584	989.06	11.7
Shared administrative support services	549,034	211.33	2.4	590,966	227.47	2.7
Total costs	22,795,748	8774.35	100.0	22,026,504	8478.25	100.0
Cost of intervention per delivery	27,203	10.47		24,420	9.40	
Incremental cost of intervention per delivery	79,427.69	30.57		41,171.03	15.85	

*UGX* Ugandan Shillings, *US* United States Dollar

## Discussion

We found that the baby-kit and the transport-voucher schemes markedly increased the service coverage of institutional deliveries at the intervention facilities over the relatively short study period. The DID analysis indicated that 30.0% of the deliveries at Ngai HC III during the intervention period were attributable to the provision of the baby kit. Ngai HC III is the only HC in Ngai sub-county. This element, combined with the HC’s proximity for the women living in the Ngai sub-county and with the baby- kit intervention, could indeed build up to a motivational effect for mothers to deliver at the facility.

The transport vouchers had a greater effect (42.9%) on the coverage of institutional deliveries when we compared the two interventions. This is a surprising finding, given that the transport vouchers were implemented at HC IIs, which have less technical capacity to conduct deliveries and that are considered to offer health services of poorer quality. Nonetheless, we believe that a combination of health system strengthening efforts with the introduction of transport vouchers may have led to positive synergies in terms of facilitating institutional deliveries.

Similarly, the service coverage of four ANC visits and at least one PNC visit increased during the interventions, with the transport vouchers demonstrating greater effect. Essentially, these outcomes are repetitive health-seeking activities that require improved access to promote utilisation. Our findings, therefore, suggest improved access to, and use of these services over time. The trends in ANC, delivery, and PNC services use by the mothers were pronounced between the transport-voucher facilities compared to their control facility than between the baby-kit and its control facility (figs. 1 and 2). Indeed, ample published literature demonstrates that the vouchers improved service utilisation and health outcomes among the target populations [54]. The demand-side financing intervention using vouchers has been associated with increased use of ANC, institutional deliveries, PNC, and reduced inequities in institutional deliveries in Pakistan [55, 56]. A study in Kenya found that some of the women who purchased vouchers meant to cover direct healthcare costs (service vouchers), did not use them because of high transport costs to the health facilities [57].

We also found that the level of ‘bypassing’ increased considerably during the study period, and it was transport-voucher-related, mainly. Although several factors may explain this finding, the unequal distribution of health facilities and the low quality of services provided by many of the facilities in the district, remain key in promoting “bypassing.” A previous study conducted in the district, and others performed elsewhere demonstrate that distance, perceived quality of care, and affordability are principal determinants of utilisation of delivery services, among others [17, 24, 58]. Hence, it is highly likely that the transport vouchers removed major barriers, particularly, distance and transport costs and encouraged pregnant women to use the facilities of their choice for maternal health services. Indeed, the DID analysis showed that the transport vouchers promoted ‘bypassing’ which in turn moderately improved service coverage in the transport voucher areas.

As illustrated above, the baby kits and factors such as proximity and a relatively higher quality of services may have attracted women from Ngai sub-county and some nearby villages to deliver at Ngai HC III, where the baby kits were implemented. Logically, the above scenario could encourage ‘bypassing’ from neighbouring communities to some extent. Notwithstanding, for women living in distant communities, the baby kits and the perceived quality of services did not appear to have had any effects on the barriers posed by long distances and the costs of transport to the centre, when compared with the effects of the transport vouchers.

Approximately 37% of the increase in pregnancy and labour-related referrals to a higher level of care was attributable to the interventions. The referrals were predominantly to Anyeke HC IV and Aber Hospital. As shown earlier, the overall facility utilisation increased with the interventions; thus, the increase in referrals might reflect the increase in the proportion of women with complications or at risk of developing complications. Considering that over 70% of the health facilities in the district are HC IIs without adequate capacities to manage complications related to pregnancy and labour, the increase in referrals is consistent with expectation. A recent study reported that although Anyeke HC IV plays a vital role in managing obstetric emergencies, only Aber Hospital has the capacity to perform all comprehensive and basic EmONC functions within the district’s health system [23]. This calls for an effective referral system that ensures a reliable and timely transfer of patients from lower level health facilities to referral centres. We believe that by strengthening the referral system through effective and free ambulance services, and free caesarean sections at Anyeke HC IV and Aber Hospital, the incentive schemes may have indirectly promoted women’s access to and use of skilled attendants during deliveries in the district [9, 27].

Of note, our interventions also resulted in unintended but largely positive effects that were predominantly associated with the transport-voucher system. From our experience, the transport-voucher system was a new concept in the district, as such it triggered excitement, and encouraged participation with the potential for some people to make small economic gains. As an illustration, some VHTs and family members or friends who owned bicycles and or motorbikes, used their knowledge of the context to sensitise households and community members about the intervention while advertising themselves as transporters.

Studies report that male involvement in maternal and newborn health services is low in Uganda [59–61]. Nevertheless, as news of the transport-voucher intervention spread through communities, some husbands and partners began to transport their spouses to the health facilities to redeem the transport vouchers. Based on the accounts of midwives and nurses responsible for the study facilities, many of the transporters were either husbands or partners of the pregnant women, who used the facilities during the intervention period. While at the health facilities, the health workers engaged most men in discussions about maternal, newborn, and reproductive health issues. Consequently, the transport vouchers seemed to have generated interest in some segments of the communities in the district, and indirectly promoted male involvement in maternal and newborn health services, to some extent. Pariyo et al. [62] reported some of these unintended effects. That study also elaborated on the potential risks and the need to enforce traffic regulations and safety precautions when using local transport mechanisms (e.g. bicycles and motorbikes) to promote maternal and newborn health services in rural settings.

### Financial costs of the interventions

The cost analysis revealed that it cost US$15.9 and US$30.6 for every additional institutional delivery attributable to the transport-voucher and baby-kit schemes, respectively. Our results imply that providing transport vouchers is less costly than baby kits in facilitating institutional deliveries. The baby kit probably has less scope for usage (reducing costs related to newborn care while encouraging facility deliveries) than the transport-voucher scheme, which facilitates the use of the institutional delivery system by addressing major barriers (distance and transport costs), to accessing facility-based services. Since health services are predominantly facility-based, and majority of the district’s population live beyond 5 km of a health facility, the transport-voucher scheme translated into having a wider impact on the study’s outcomes.

The two incentive schemes were implemented simultaneously to improve access to and use of maternal and newborn healthcare services in the district. The resultant effects were notable and indicated which intervention to prioritise in the event of budgetary constraints.

As of yet, there are few publications on the cost-analysis of transport vouchers and hardly any research literature on baby kits, which makes this study a significant contribution to the literature. Moreover, regardless of the lack of comparable literature, in a setting where more than half of the population lives below the poverty level [20], our interventions may not be affordable or sustainable without substantially more public-health funding from the government and development or implementing partners.

### Study limitations

We acknowledge several limitations to our study that we think might be related to the setting and methodological challenges posed by implementing a study within a larger programme. The control and intervention groups were not randomly selected, which could lead to possible biases. As described in the Methods, the intervention facilities were purposively selected based on their poor performance to improve the maternal and newborn health services in the affected sub-counties. Nonetheless, their controls were comparable. Furthermore, the use of aggregated health-facility data did not allow the use of statistical analyses to adjust for confounders and to account for uncertainty in our estimates by calculating 95% confidence intervals. We are, however, confident that the DID analysis enabled us to demonstrate the effects of the interventions on the study outcomes, given the limitations.

We think that some deliveries that could be attributed to the interventions, particularly the transport vouchers, may have occurred at health facilities outside our study catchment areas. As such, those deliveries were not included in the analysis according to our study design. This means that we might not have captured the full effects of the interventions regarding institutional deliveries.

Likewise, the cost analysis did not capture the effects of the interventions on other services, such as changes in the uptake of ANC and PNC services. Consequently, the analysis probably underestimated the full effects of each intervention.

The district-wide measures to strengthen the health system were congruent with the need for a stronger health system to implement the interventions and achieve the study outcomes [63, 64]. Notwithstanding, some of the measures might have resulted in synergies that are desirable; yet, they might also confound some of the effects of the interventions, especially the effects on referrals and ‘bypassing’.

The overlap of the catchment areas of the health facilities or the boundaries of the study’s sub-counties could have led to over or underestimation of the calculated percentage of ‘bypassing’. Although we made efforts to minimise this phenomenon, the quest for better quality of care and affordable services might have been a distraction from some of those efforts. Considering these methodological challenges, we urge caution in interpreting our findings. Additionally, we think that a qualitative study on these interventions could complement our findings.

We also recognise that this study was conducted in a rural post-conflict district in northern Uganda, where the context might not permit generalisation of our findings to other settings. Despite the limitations, we firmly believe that our findings remain valid as well as relevant.

## Conclusions

Implementation of a transport voucher scheme in Oyam District effectively increased the utilisation of ANC, institutional childbirth, and PNC services whereas the baby-kit scheme increased utilisation of institutional delivery. Both interventions had a positive effect on referrals. ‘Bypassing’ increased and it was essentially transport voucher-related.

The transport-voucher system was less costly than providing baby kits in facilitating institutional deliveries. The finding that the provision of transport vouchers was less costly and had a wider influence, including unintended but largely positive effects suggests that in the event of budgetary constraints, the preference would be to use the transport-voucher system. We believe these incentives can be sustainable if the Ministry of Health budgets for and integrates them in the health system, especially in underserved areas.

## Additional files

Additional file 1: Details of cost items analysed. This table shows the different cost items analysed for the transport voucher and baby-kit schemes. (DOCX 14 kb)

Additional file 2: Number of pregnancy and labour-related referrals. This table shows the number of women referred from the intervention and control health facilities to the hospital or the Health Centre IV. (DOCX 14 kb)

## Abbreviations

ANC: Antenatal care
DID: Difference in differences
EmONC: Emergency obstetric and neonatal care
HC: Health centre
HSD: Health sub-district
MDGs: Millennium development goals
MMR: Maternal mortality ratio
NGO: Non-governmental organisation
OPD: Out patients’ department
PNC: Postnatal care
PNFP: Private not-for-profit
SSA: Sub-Saharan Africa
VHT: Village health team
