# Supplementary material for: Effects of demand-side incentives in improving the utilisation of delivery services in Oyam District in northern Uganda: a quasi-experimental study
Source: BMC Pregnancy Childbirth. 2017 Dec 19;17:431. doi: 10.1186/s12884-017-1623-y (PMC5737523; doi:10.1186/s12884-017-1623-y)
Supplement: Supplementary file 1 — Details of cost items analysed. This table shows the different cost items analysed for the transport voucher and baby-kit schemes. (DOCX 14 kb) [file 12884_2017_1623_MOESM1_ESM.docx]

**Additional file 1: Details of cost items analysed**

| **Costs** | **Transport Vouchers** | **Baby Kit** |
| --- | --- | --- |
| **Direct costs** | - Amount redeemed by transporters of women - Vouchers distribution, payment of riders, and collection of voucher receipts. Includes fuel and vehicle maintenance costs - Printing of transport vouchers | - Total cost of materials purchased for baby kits - Printing of receipts for the baby kits - Cost of cutting and packing materials for baby kits - Transporting baby kits from Aber to Ngai |
| **Sensitisation costs** | - Airtime for FM Radio talk shows - Allowance for talk show participants - Cost of radio spot messages - Stakeholder sensitisation meetings | - Airtime for FM Radio talk shows - Allowance for talk show participants - Cost of radio spot messages - Stakeholder sensitisation meetings |
| **Training costs** | - Meals, transport refund, daily allowance for facilitators, training hall hire, stationery | - Meals, transport refund, daily allowance for facilitators, training hall hire, stationery |
| **Labour costs** | - Social worker's salary (for time spent on project). Includes supervision costs - Drivers salary (for time spent on project) | - Social worker's salary (for time spent on project). Includes supervision costs - Drivers salary (for time spent on project) |
| **Shared administrative support services** | - Driver allowances - Transportation costs from Kampala to Oyam District | - Driver allowances - Transportation costs from Kampala to Oyam District |
