# Supplementary material for: Effects of demand-side incentives in improving the utilisation of delivery services in Oyam District in northern Uganda: a quasi-experimental study
Source: BMC Pregnancy Childbirth. 2017 Dec 19;17:431. doi: 10.1186/s12884-017-1623-y (PMC5737523; doi:10.1186/s12884-017-1623-y)
Supplement: Supplementary file 2 — Number of pregnancy and labour-related referrals. This table shows the number of women referred from the intervention and control health facilities to the hospital or the Health Centre IV. (DOCX 14 kb) [file 12884_2017_1623_MOESM2_ESM.docx]

**Additional File 2: Number of pregnancy and labour-related referrals**

| **Name of health facility** | | **Facility type (level)** | **Baseline value (2013)** | **Endline value (2014)** |
| --- | --- | --- | --- | --- |
| Baby kit intervention | |  |  |  |
|  | Agulurude (control) | Health Centre III | 23 | 34 |
|  | Ngai | Health Centre III | 31 | 50 |
| Transport voucher intervention | |  |  |  |
|  | Amwa (control) | Health Centre II | 11 | 18 |
|  | Atipe + Alao | Health Centre IIs | 26 | 46 |
